# The adaptation of meaning centered psychotherapy to develop RELOAD-C: a web-based tool to reduce loneliness in caregivers of persons with Alzheimer’s disease and related dementias

**DOI:** 10.1093/tbm/ibaf059

**Published:** 2025-01-16

**Authors:** Allison Marziliano, Huma Babar, Priya Patel, Marzena Gieniusz, Jessica Mongelli, Edith Burns, Allison J. Applebaum, Hayley Pessin, William Breitbart, Liron Sinvani, Carla Perissinotto, Michael A. Diefenbach

**Affiliations:** 1 Institute of Health System Science, The Feinstein Institutes for Medical Research, Northwell Health, Manhasset, NY, United States; 2 Department of Medicine, Northwell Health, The Donald and Barbara Zucker School of Medicine at Hofstra/Northwell, Hempstead, NY, United States; 3 The Alzheimer’s and Dementia Care Program, Northwell Health, Manhasset, NY, United States; 4 Department of Psychiatry and Behavioral Sciences, Memorial Sloan Kettering Cancer Center, New York, NY, United States; 5 Department of Medicine, Division of Geriatrics, University of California, San Francisco, CA, United States

**Keywords:** loneliness, meaning, purpose, caregiver, dementia, intervention

## Abstract

**Background:**

More than 60% of caregivers of persons with Alzheimer’s disease and related dementias (AD/ADRD) are lonely. Meaning and purpose in life is associated with reduced feelings of loneliness, but has not yet been systematically fostered among caregivers of patients with AD/ADRD. Adapting meaning-centered psychotherapy (MCP), an evidence-based treatment for increasing meaning and purpose in life in cancer caregivers, might decrease loneliness in the dementia caregiver population.

**Purpose:**

The purpose of this manuscript is to report on the development, usability, and acceptability testing of REducing LOneliness in Alzeheimer’s Disease-Care Partners (RELOAD-C), a web-based platform that features six brief videos and aims to reduce loneliness in caregivers of patients with dementia through introducing major concepts and principles adapted from meaning centered psychotherapy.

**Method:**

Within 12 months, RELOAD-C was developed through two rounds of one-on-one interviews with 15 dementia caregivers to obtain feedback on video scripts, recording of videos, and placement of videos and written content (e.g. thought exercises) on the website. Following this, RELOAD-C underwent rigorous usability and acceptability testing by another 16 dementia caregivers.

**Results:**

Quantitative assessments show that RELOAD-C is deemed usable by caregivers (mean = 1.69 on system usability scale, where possible range is 1–5 and lower scores indicate more favorable views of the website; and more than 90% of the usability sample correctly engaged in ≥8 of 10 discreet tasks). Qualitative data indicate acceptability of the intervention with feedback such as “love that the videos are clear and load fast.”

**Conclusions:**

RELOAD-C is a web-based intervention focused on reducing loneliness in dementia caregivers. It contains six therapist-narrated videos and written content, reinforcing MCP principles. It is currently undergoing pilot testing in preparation for a large-scale randomized controlled clinical trial evaluating its efficacy in reducing loneliness in dementia caregivers.

## Lay Summary

More than 60% of caregivers of persons with dementia are lonely. Loneliness is associated with many negative health outcomes, including increased feelings of burden and poor quality of relationships. Adapting meaning-centered psychotherapy, a treatment for increasing meaning and purpose in life in cancer caregivers, might decrease loneliness for dementia caregivers given the inverse relationship between meaning/purpose in life and loneliness. The purpose of this study is to report on the development and usability and acceptability testing of REducing LOneliness in Alzeheimer’s Disease-Care Partners (RELOAD-C), a website that aims to reduce loneliness in dementia caregivers through adapting major concepts and principles of meaning centered psychotherapy. Over the course of 12 months, RELOAD-C was developed through two rounds of one-on-one interviews with 15 dementia caregivers to obtain feedback on video scripts, recording of six brief videos, and placement of videos and written content (e.g. thought exercises) on the website. Following this, RELOAD-C underwent usability and acceptability testing by another 16 dementia caregivers, who indicated that RELOAD-C is acceptable to caregivers and easy to use. The website is currently being evaluated in a pilot trial in preparation for a large-scale randomized controlled clinical trial.

## Introduction

Globally, more than 55 million people have Alzheimer’s disease and related dementias (AD/ADRD). There are nearly 10 million new cases of AD/ADRD annually, drastically increasing the number of caregivers each year [1]. According to the Alzheimer’s Association, in the United States alone, over 11 million individuals provide unpaid care for people with AD/ADRD [2]. Although there are a range of definitions for the term “caregiver,” our focus is on those who tend to the needs or concerns of a loved one, family member, or friend, and does not include those who are caring for another person as part of their primary employment [3] (i.e. nurses, home health aides). More than 60% of caregivers of persons with AD/ADRD report experiencing loneliness [4], defined as the distressing experience when one’s relationships are poorer in quantity and quality than desired [5]. This is not surprising given the unique experience of caring for a person with AD/ADRD, characterized by a decline in intellectually stimulating conversation, loss of mutual support, and fewer social outings.

In the general population, loneliness is associated with a 26% increased risk of mortality, and physical and mental morbidity [6]. In longitudinal studies, loneliness significantly predicts heart attack [7], diabetes [8], depression, anxiety [9, 10], and distress [11]. Studies of caregivers show that loneliness is associated with poor quality of relationships [12] and increased feelings of being burdened [13, 14]. Therefore, there is an urgent need for effective evidence-based interventions to reduce loneliness in caregivers of persons with AD/ADRD.

Existing interventions for AD/ADRD caregivers focus on providing education, decision support, skills training, and stress management; however, these have been less successful in significantly reducing loneliness over time. A qualitative analysis of 119 loneliness interventions demonstrated that their limited efficacy may be due to a lack of content focused on meaning in life [15]. This is supported by several empirical studies [16-20] which indicate a strong inverse relationship between meaning and loneliness. Macia *et al.* found that meaning in life was the most important predictor of loneliness, and the authors recommend targeting meaning in life in future interventions [19]. Folker *et al.* theorized that meaning in life promotes a better ability to cope with loneliness [20]. Existing interventions have not attempted to reduce loneliness through increasing AD/ADRD caregivers’ sense of meaning and purpose in life, despite this strong evidence that increased meaning in life predicts reduced loneliness [21-24].

Thus, interventions to reduce loneliness in AD/ADRD caregivers may be improved by incorporating concepts from meaning-centered psychotherapy (MCP), an evidence-based treatment for increasing meaning and purpose in life. MCP focuses on exploring sources of meaning and is based on the premise that finding meaning and purpose in one’s existence is a primary force of motivation. MCP was initially developed in both individual [25] and group formats [26] for patients with advanced life-limiting cancers, and has since been adapted for various populations (such as Latino [27] or Chinese Immigrant [28] patients with cancer, and individuals receiving palliative care [29]), including cancer caregivers [30]. Indeed, an examination into the mechanism of MCP confirmed hypothesized mediation effects via a sense of increased meaning and purpose in life on outcomes such as improved quality of life, and decreased depression, hopelessness and a desire for hastened death [31] among patients with advanced cancers. Yet, MCP has not been used in an AD/ADRD caregiver population, nor has the impact of MCP on loneliness in any population been systematically evaluated.

Therefore, to address loneliness in caregivers of patients with AD/ADRD, we adapted MCP for this unique caregiver population, and developed RELOAD-C (REducing LOneliness in Alzeheimer’s Disease-Care Partners). RELOAD-C is a web-based platform that explains the elements and principles of MCP through six brief videos presented by an MCP therapist and licensed clinical psychologist. The videos are an adaptation of MCP principles that were developed for cancer caregivers as a self-administered workshop [30]. The use of these videos to target loneliness is based on two premises. First, the content of the videos emphasizes MCP, which is proven effective in increasing meaning and purpose in life [31]. Given the inverse relationship between meaning and purpose in life and loneliness [16-20], we hypothesize that increasing caregivers’ meaning has the potential to reduce their loneliness. Second, the format of RELOAD-C—as a website with self-administered videos, allows MCP content to be delivered to AD/ADRD caregivers when it is convenient for them and potentially at a time when they are lonely, on their own, engaged in caregiving tasks, and in need of inspiration or guidance from a reminder of the MCP principles. In addition, RELOAD-C contains written content providing guidance on homework and exercises referenced in the videos.

The purpose of this manuscript is to report on the development, usability, and acceptability testing of RELOAD-C. The current work is preparatory for a pilot trial [32] evaluating the feasibility of our approach to conducting a large-scale randomized controlled trial (RCT). The large-scale RCT will compare three arms: (i) usual care/resources when participants report at least mild burden; (ii) RELOAD-C with MCP videos alone; or (iii) RELOAD-C with MCP videos plus 7 virtual discussion groups consisting of AD/ADRD caregivers and facilitated by a licensed social worker trained in MCP. The primary outcome is a reduction in loneliness from preintervention to 6-weeks post the start of the intervention in AD/ADRD caregivers.

## Methods

**Figure 1:**
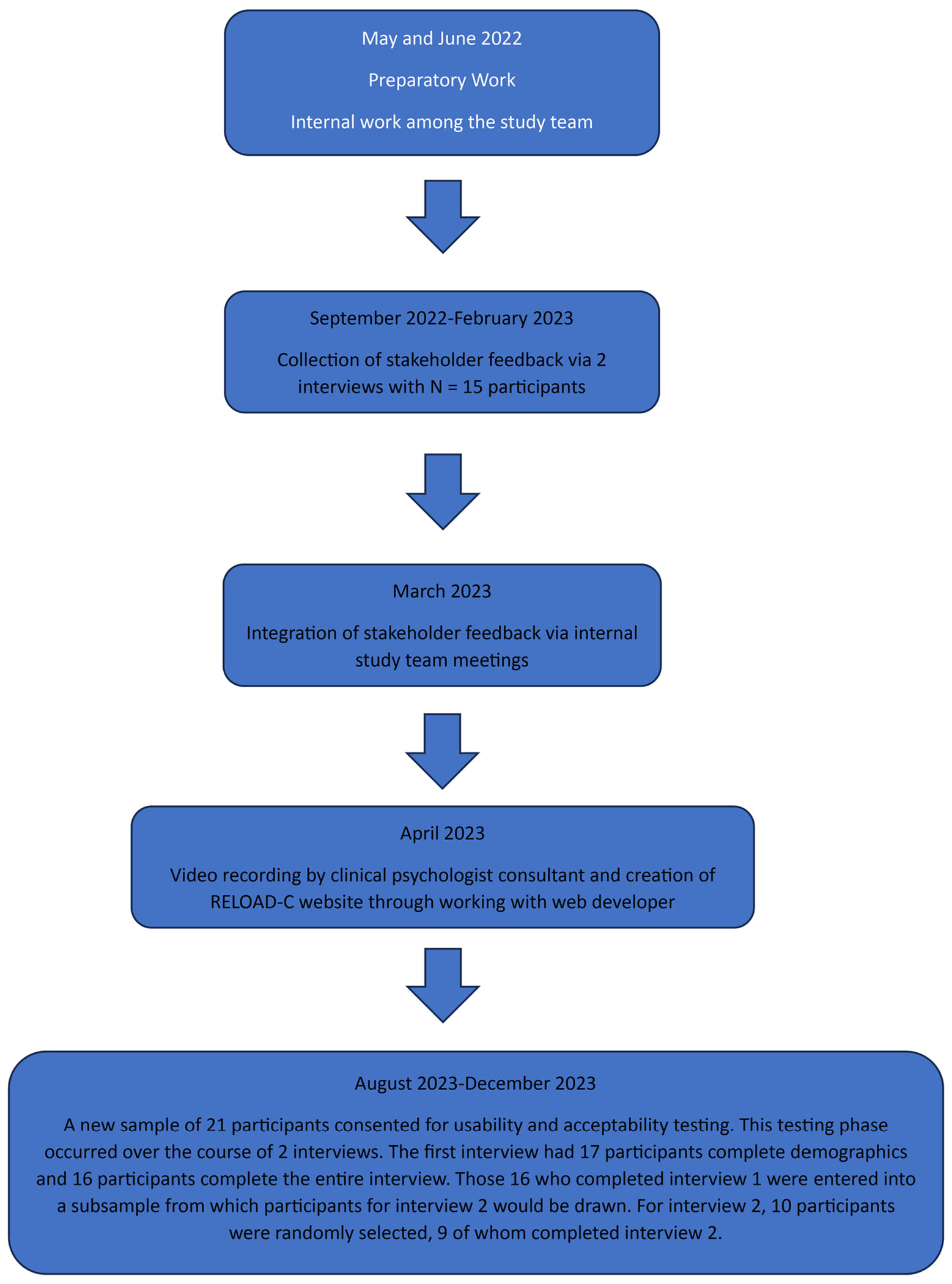
Five phases of development, usability and acceptability testing of RELOAD-C.

The development of RELOAD-C occurred during five phases: (i) preparatory work; (ii) collection of stakeholder feedback; (iii) integrating stakeholder feedback; (iv) video recording and creation of the website; and (v) usability and acceptability testing. See Fig. 1 for further details and each phase is described in detail below. This study follows a mixed methods approach to data collection in an effort to leverage the strengths of both quantitative and qualitative data. Specifically, phase 2 (collection of stakeholder feedback) and phase 5 (acceptability testing only) relied heavily on qualitative data to uncover the thoughts and feedback of research participants. Further, phase 5 (usability testing only) relied heavily on quantitative data to make it possible to determine that our website is indeed usable. All other phases (1, 3, and 4) did not involve collection of data from research participants, but were instead achieved through discussions and effort among members of the study team.

### Preparatory work (May and June, 2022)

Members of the study team conducted preparatory work consisting of reviewing transcripts of six existing videos of MCP for cancer caregivers, and editing the language to suit an AD/ADRD caregiver population.

### Collection of stakeholder feedback (September 2022 to February 2023)

After obtaining approval from the Institutional Review Board (#21-1264), a total of 15 AD/ADRD caregivers were recruited and provided informed consent. Inclusion criteria consisted of: (i) self-identify as the primary caregiver of a community-dwelling person with AD/ADRD (diagnosis confirmed via the patients’ electronic health record); (ii) affirm that their caregiver role is not related to their primary employment (i.e. they are a family member or friend of the patient) or that any financial gains for their caregiving come via Managed Long-Term Care; (iii) aged 18 or older; (iv) English-speaking; (v) cognitive competency to participate, as determined by the consenting professional; and (vi) access to a telephone, computer, Internet, and email. Of note, only one caregiver per patient was allowed to participate. In cases where there were multiple interested caregivers for one patient, the caregivers decided who among them should participate. All participants for the development phase of this study were recruited from the practice of one physician and one nurse practitioner from the Alzheimer’s and Dementia Care Program of a large healthcare system in a suburban area of New York. Research assistants screened the medical records of patients with potentially eligible caregivers coming into the clinic each week, emailed the clinicians for review/approval to approach the caregiver, and if approved, contacted the caregiver either in person at the patient’s appointment or via telephone. At the time of recruitment, after consent, participants received the video scripts and were asked to review them ahead of the study interviews.

Study procedures consisted of two interviews between the participant and the study PI (author Marziliano), conducted via telephone, each lasting between 20 and 30 minutes. The average number of days between consent and interview 1 was 8 days, and the average number of days between interviews 1 and 2 was 9–10 days. The study team aimed for about 1 week between the time of consent, during which the videos scripts were provided to participants, and interview 1 in the hopes that this would be sufficient time for participants to review all of the materials in detail and make notes of their feedback, but not too much time that they would misplace the materials or forget about the study altogether. For a similar reason, we aimed for about 1 week between interviews 1 and 2 to collect any additional feedback that the participants may have thought of after their first conversation with us.

During the first interview, the study PI (author Marziliano) collected demographic data: name, gender, race, ethnicity, marital status, relationship to the patient, time since becoming a caregiver in months, address, phone number, email, and date of birth. Additionally, the PI (author Marziliano) collected general thoughts and feedback on the video scripts, using the prompt: “Please share with us how reading these scripts makes you feel, how they resonate with you, words that you like or don’t like, and what more you would like.” Separate from the general feedback, the PI (author Marziliano) also asked specific questions about the video scripts, such as “What term do you prefer to describe your role? (caregiver, care partner, career)” (see Table 1). During the second interview, occurring about 1 week after the first interview, participants were prompted to give any additional thoughts on the scripts after having had some time to digest the content. Research personnel typed the participants’ responses into REDCAP as they spoke.

In terms of the sample characteristics, *N* = 15 AD/ADRD caregivers completed two one-on-one interviews with the study PI (AM). Most caregivers were female (*n* = 10), White (*n* = 11), non-Hispanic (*n* = 12), and married/partnered (*n* = 12). About half were caring for a spouse (*n* = 8) while the rest cared for a parent (*n* = 7). On average, caregivers were 61.67 years old and had been providing care to their loved one for 3.75 years.

### Integrating stakeholder feedback (March 2023)

The study PI (author Marziliano) reviewed feedback from both interviews of all 15 participants and presented a summary of their feedback to the study team in a series of meetings.

### Video recording and creation of the RELOAD-C website (April 2023)

The developer of MCP for Cancer Caregivers (MCP-C) [30] is a consultant on this study and a co-author on this manuscript (author Applebaum). This author video-recorded herself in six brief videos, each of which portrayed her speaking the text in the six video scripts that had been reviewed and refined by the sample of 15 stakeholders. While the first and last videos are dedicated to an introduction to the intervention and a conclusion/wrap up, respectively, the four videos placed in the middle each unravel one of the four sources of meaning: historical, attitudinal, creative and experiential. The PI (author Marziliano) simultaneously worked with a web developer to create RELOAD-C, a web-based platform that houses these six videos, as well as written content on MCP. Of note, the written content on the RELOAD-C website is merely thought exercises, questions, and prompts that are already part of the video scripts and therefore was reviewed by stakeholders within the confines of the video scripts. We used the MCP written content as a way to reinforce some of the thought questions discussed in the videos. We hope that having them there in text on the RELOAD-C website encourages people to think about these questions both before and after they view the related video.

### Usability and acceptability testing of RELOAD-C (August–December 2023)

Following integration of the stakeholder feedback, recording of the six brief videos, and development of the website, 21 new AD/ADRD caregivers were recruited and consented to participate in usability and acceptability testing of RELOAD-C, which occurred during two separate interviews. This occurred after obtaining approval from the Institutional Review Board (#21-1264) and securing informed consent from the research participants. Caregiver recruitment and eligibility for this usability and acceptability phase were identical to the stakeholder feedback phase. Of note, participants from the first phase were excluded from this phase. Participants recruited for the usability and acceptability phase of this study were also recruited from clinicians (one physician and one nurse practitioner) from the Alzheimer’s and Dementia Care Program of a large healthcare system in a suburban area of New York. For all aspects of this aim (demographics, 10 discreet tasks, system usability scale (SUS), qualitative data from the Think Aloud technique), the research personnel typed the participants’ responses into REDCAP as they spoke.

In sum, 21 caregivers were consented and invited to participate in interview 1 of the usability and acceptability session. Of the new and distinct sample of 21 caregivers who signed consent for the usability and acceptability testing, one withdrew soon after consent, another one was unable to share her screen via Teams and therefore could not participate, and two more caregivers were lost to follow-up, leaving 17 caregivers as our sample for this part of the study. One of the 17 participants completed only the demographic assessments, but not the rest of the study, leaving 16 participants with complete data. See Table 2 for their demographics.

A random number generator was used to select 10 participants who completed interview 1 for a second interview. Although 10 participants who completed interview 1 were re-contacted to participate in interview 2, 9 agreed/consented to participate in interview 2. One participant was unable to be reached.

#### Usability testing

During interview 1 of the session, the participant joined a virtual meeting via Microsoft Teams; provided demographics; opened the RELOAD-C website on their computer; and shared their screen via Teams. To assess usability, the participant was asked to navigate through 10 discreet tasks during a Think Aloud session (see Table 3); and completed a 10-item SUS (see Table 4). Sample items from the standardized, validated 10-item SUS [33] are: “I think that I would like to use this website frequently” and “I found the website unnecessarily complex.” All 10 items were rated on a 5-point Likert scale ranging from 1 = strongly agree to 5 = strongly disagree. During interview 2, participants were asked questions related to comfort and usability of Microsoft Teams (see Table 5). Given our institution’s approval of Microsoft Teams, we selected to use Teams as the video conferencing program to facilitate our virtual evaluation of the usability of RELOAD-C. However, we also thought it was important and an ideal time to collect data during this phase on whether Teams is the appropriate video conferencing program to use in our upcoming pilot trial and what Teams features were deemed usable by the study participants.

#### Acceptability testing

To assess acceptability, during the entire interview 1, participants were reminded to state their thoughts aloud (i.e. the Think Aloud technique). During interview 2, additional qualitative feedback was collected, particularly on the acceptability of the changes made to the website after the first interview.

### Statistical analyses

In terms of data analyses, during the Phase 1 preparatory work, frequent meetings were held among the study team members to discuss the content of the original scripts and changes were made when more than one study team member was in favor. The analysis of the Phase 2 data collection occurred during Phase 3 (integration of stakeholder feedback). During Phase 3, demographic data was summarized using means and standard deviations for continuous variables and frequency and percentages for categorical variables. The feedback from both interviews of all 15 participants in Phase 2 was reviewed by the Principal Investigator (author Marziliano) and presented in summary form to the study team in a series of meetings. The study team collectively decided which feedback to integrate into the scripts, with feedback referenced more than one time as a guideline for inclusion. Phase 4 did not involve data collection but was rather an iterative conversation among the study team, the clinical psychologist consultant who video-recorded herself delivering the scripts, and the web developer. Lastly, for phase 5’s usability testing, demographics were summarized using means and standard deviations for continuous variables and frequency and percentages for categorical variables. For the administration of the 10 discreet tasks, study personnel watched participants’ performance via Microsoft Teams and recorded whether they were able to complete the task without assistance. Frequencies and percentages were identified for the following categories: completed all 10 tasks, completed 9 of the 10 tasks, completed 8 of the 10 tasks, and completed 7 of the 10 tasks. For the SUS, negatively worded items 2, 4, 6, 8, and 10 were reverse coded and means were calculated across all participants for each of the 10 items, with lower means indicating more favorable usability of RELOAD-C. For the questions regarding the use of Teams, the study personnel selected the most appropriate category (on their own/no help needed, able to complete with help, could not complete even with help) for each question (joining Teams, raising hand function, turning on/off microphone) to describe the research participants’ use of Teams and data was summarized using frequencies and percentages. For phase 5’s acceptability testing, qualitative data from the Think Aloud Method was analyzed in a similar manner as the data from phase 2. Specifically, the feedback was reviewed by the Principal Investigator (author Marziliano) and presented in summary form to the study team in a series of meetings. If facets of the website were reviewed unfavorably by more than one participant, discussions took place among the team about making a change.

## Results

The results of each phase of the development process are outlined below, organized as: (i) preparatory work results; (ii) Results from the collection of stakeholder feedback; (iii and iv). integrating stakeholder feedback results, video recording, and creation of the website; and (v) usability and acceptability testing results.

### Preparatory work results

Changes made to the transcripts at this stage included replacing the word “cancer” with “dementia,” and removing the exercises or homework that would require high-level discussion between the caregiver and a cognitively intact patient.

### Results from the stakeholder feedback

Participants provided meaningful general feedback (see Table 6 responses) on the video scripts during both interview rounds, much of which was integrated into the final scripts. Examples include suggestions to add practical advice for caregivers, defining key terms from MCP such as “experiential,” and increasing reference to loneliness throughout the videos.

However, some feedback, while noteworthy, was not integrated into the video scripts, as making such substantial changes could have altered the core elements of MCP, which have been already proven effective in increasing meaning and purpose in life in cancer caregivers. Examples of these recommendations from caregivers are: (i) removing quotes or references to Victor Frankl, the Holocaust, the exit visas, or concentration camps; (ii) changing the order in which the sources of meaning are presented; and (ii) increasing or decreasing the time spent on a specific source of meaning.

Caregivers also provided feedback on the social worker’s script (see Table 7 responses). Themes for these responses highlighted the terminology preferred when referring to the group meetings, as well as providing greater detail to participants about the logistics of the group during the first meeting.

In addition to providing general feedback on the video and social worker’s scripts, caregivers also responded to five specific questions (see Table 1 responses). Examples of these responses include preference for the term “caregiver” rather than “care partner,” use of the term “dementia” to describe the illness that their loved one has, and recommendations that the study team define what is meant by “unfinished business” and provide examples for the participant. Of note, the responses provided to all of these questions were used to make changes to the intervention.

All 15 participants were reconducted for a second interview with the study PI (author Marziliano) to give any final thoughts on the scripts; however, no substantial feedback emanated from these second interviews.

### Integrating stakeholder feedback results, video-recording and creation of the website (March–April 2023)

Feedback from these interviews was integrated into the scripts and the six brief videos were recorded by our MCP expert, author Applebaum. We sent the videos to our web developer who inserted them into the RELOAD-C website. Several refinements were made to improve the videos’ clarity and placement, as well as the text, color scheme and layout of the RELOAD-C website. The six videos were, on average, 7.3 minutes long (Video 1: 13 minutes; Video 2: 8 minutes; Video 3: 6 minutes; Video 4: 8 minutes; Video 5: 4 minutes; and Video 6: 5 minutes).

### Usability and acceptability testing results (August–December 2023)

#### Usability testing

During interview 1, 16 of the 17 caregivers completed the 10 discreet tasks (see Table 3 for tasks) on the RELOAD-C website while the study personnel watched their performance via Microsoft Teams and recorded whether they were able to complete the task without assistance. Of the 16 caregivers, *n* = 13 (81.3%) correctly completed all 10 tasks, one person (6.25%) correctly completed 9 of the 10 tasks, one person (6.25%) correctly completed 8 of the 10 tasks, and one person (6.25%) correctly completed 7 of the 10 tasks.

Also during interview 1, 16 of the 17 caregivers completed the SUS. The mean SUS score across all 10 items was 1.69 (possible range is 1–5), where lower scores indicate more favorable views of the website. See Table 4 for results.

About half of the participants were able to use Microsoft Teams with ease and on their own; the other half needed assistance from the research staff or were unable to join Teams or share their screens even with assistance. In these cases, research personnel met the study participant in person at their upcoming clinic appointment to conduct the testing session in person. The difficulty using Microsoft Teams prompted our team to add on questions specifically about comfort with and usability of Teams during interview 2. The subgroup who completed interview 2 was relatively comfortable with Teams (see Table 5 for results on questions regarding the use of Microsoft Teams).

#### Acceptability testing

During interview 1, as part of the Think Aloud method, qualitative data were also collected. Most of the comments were very positive regarding the website. Sample comments regarding the website were: “I like the side bar,” “the website is easy to navigate,” “the videos load right away which is very nice,” “love that the videos are clear and load fast.” In a few cases, participants commented on the pale colors used in the website and suggested we use more vibrant colors, which prompted us to add bright scenic pictures on many of the website pages (e.g. scenes of a sunset, ocean, flowers, and mountains).

During interview 2, in addition to the quantitative usability questions related to comfort with Teams, we also asked participants to again give their open-ended feedback on both the acceptability of the website and Teams, particularly on changes stemming from comments collected during interview 1. Regarding the final version of the website, participants indicated that it “looked great” and was “very flushed out with more detail.” One participant noted that she really appreciated that the videos appear bit by bit (one video appears weekly) so it is not too much to digest at once. Caregivers appreciated the bullet point format. They also appreciated that the transcript of the videos appeared at the bottom of the website as the videos played. Others referred to the website as “clean, straightforward and easy to navigate,” and asked when the website would be made available to the public. One caregiver stated that having a wife with AD/ADRD has been very difficult and he would have liked to have this as a tool for support. One participant indicated that he was grateful that the website encouraged him to self-reflect and take time to pause over the course of the day and reflect on his past, present, and the big picture in life.

While some additional feedback could not be integrated into this version of RELOAD-C, it will be considered for future iterations of the website. This includes adding names and contact information of relevant healthcare professionals in the area and offering the videos and written content in languages other than English.

As a result of these efforts, which included four rounds of review by at least 35 different dementia caregivers over the course of 1.5 years, the website, RELOAD-C, was created and deemed usable and acceptable. See Table 8 for details about each of the 6 MCP videos that are on the RELOAD-C website.

## Discussion

This manuscript details the process of developing, and assessing the usability and acceptability of RELOAD-C. RELOAD-C is a web-based intervention that aims to reduce loneliness in dementia caregivers. RELOAD-C houses six brief MCP videos and written content reinforcing the concepts presented in the videos. This is the first support tool for caregivers of persons with AD/ADRD that focuses on MCP principles. The development process, which occurred over the course of nearly 1 year, involved multiple rounds of review and integration of feedback by the study team, experts in the field of dementia, and dementia caregivers themselves. In addition, development of this web-based intervention relied on input from consultants with expertise in MCP and a team of web developers. Once created, the study team spent another 5 months putting RELOAD-C through assessments for usability and acceptability, which, again, spanned multiple rounds of review with a new set of 16 dementia caregivers. During this phase, not only was RELOAD-C evaluated, but so too was the ease of using virtual conferencing modalities (i.e. Microsoft Teams) in preparation for the pilot trial [32] to assess the feasibility of our approach to conducting a large-scale RCT. The feedback provided indicated that caregivers, after multiple rounds of refinement, found the format, platform, and content of the RELOAD-C intervention both usable and acceptable.

### Study contributions and implications for the field

This study forges beyond reducing loneliness in dementia caregivers in two ways. First, the process of intervention development, usability testing, and acceptability testing, can serve as a model for future web-based interventions of various types, with various intents, and for various populations. Having this established model for development provides a map for other researchers to follow. Second, given that loneliness is a prominent public health problem affecting many different populations, if shown to be effective in follow-up studies, this web-based intervention can be easily adapted for other groups impacted by loneliness.

### Strengths and limitations

This study has several strengths. First, in order to develop this web-based intervention, the study team combined and integrated perspectives from multiple individuals, all of whom have expertise in the population of interest. Most importantly, the study team collected feedback from dementia caregivers themselves. Second, not only were multiple perspectives involved in the development of this website, but there were also multiple rounds of review, allowing participants time to digest the material and offer both immediate and non-immediate feedback. Third, we felt our goal to recruit a diverse sample was achieved, particularly for the 21 caregivers for the usability and acceptability session. Nearly a quarter of the participants report Black/African American as their race and 17.7% report Asian/Pacific Islander as their race. In terms of relationship to the patient, we had participants with various relationships. Most were daughters of the patient, but we also had spouses, close family friends, and granddaughters of the patient represented in the data. As with any study, we did meet limitations. First, we didn’t have the time and resources available to engage in a formal thematic analysis of the qualitative data, but we felt this was not necessary given the narrow purpose of this study being to develop the web-based intervention. Second, our recruitment team consisted of English-speaking research assistants only, hindering our ability to recruit participants who did not speak or read English.

### Future directions

Following the development and usability and acceptability testing of RELOAD-C, the next step, which is currently ongoing, is a pilot trial [32] to evaluate the feasibility of conducting a large-scale RCT. Following conclusion of the pilot trial, subsequent studies will aim to advance our intervention, RELOAD-C, through further stages of the National Institutes of Health (NIH) Stage Model for Behavioral Intervention Development. These stages are: (i) under a planned R01, conduct a large-scale RCT to evaluate efficacy of RELOAD-C components in reducing loneliness in care partners; (ii) conduct a RCT with community-based care partners evaluating efficacy-effectiveness of RELOAD-C components in reducing loneliness; (iii) examining RELOAD-C components in community settings with community care partners while maximizing external validity; and (iv) examining strategies of implementation of RELOAD-C components in community settings.

## Figures and Tables

**Table 1:** Caregivers’ responses to specific questions (*N* = 15)

Questions	Theme (if applicable)	Examples of responses
1.What term do you prefer to describe your role?	-	Nearly all (13/15, 86.67%) said “caregiver” citing that this was the term they had heard most often. One participant who did not prefer the term “care partner” indicated that she does everything for her loved one, and to refer to her as only a “partner” diminishes her role.
2. What term do you prefer to refer to the illness your loved one has?	-	The most common response was “dementia” (12/15, 80%). One caregiver said that terminology depended on who she was talking to, such that she used the umbrella term “dementia” most often with friends and family, but specified “Alzheimer’s Disease” when talking to physicians.
3. What do you think about our reference to the book “Man’s Search for Meaning” during Video 1?	Mixed feedback	Caregivers appreciated the opportunity to seek out the book to learn more about what is summarized in the videos. However, the thought of reading an entire book as yet another task for a caregiver could be quite daunting, so they recommended that our team stress reading the book is optional and it can be rented from the local library or is available online free of charge. One caregiver brought up the idea of using audiobooks, so she could listen to it while she engages in other tasks since there is little time to sit down and read a book. Other caregivers noted ways to increase the likelihood that participants will read the book, such as showing a copy of the book in the video, saying how long it is, or including more excerpts from the book in the videos.
4. Referring to the example in Video 3, in which the caregiver and patient repair a relationship through high-level, rationale discussion, is this appropriate to leave in?	Removal of irrelevant examples	Most participants expressed that this example was not relevant for their situation, and that such meaningful conversations could not be accomplished beyond the earlier stages of AD/ADRD. Another caregiver said that this type of example is too complex to be mentioned in passing during a brief video. Some caregivers indicated that their loved ones are exhibiting behavioral and psychological symptoms of AD/ADRD and are angry and irritable, while others said that their loved one has the cognitive functioning of a child, cannot hold a conversation, or has the same few conversations over and over. Some caregivers said that their loved one’s reasoning is completely absent, and that they avoid talking with their loved one about problems because he or she becomes nervous or upset.
5.What do you think about the language in Video 4 refencing “unfinished business”?	Confusion	Caregivers’ varied responses made it clear that this term means something different to each person. Whereas some thought of unfinished business as activities the caregiver was engaged in but had to put on hold so they could care for their loved one, others thought of unfinished business as events he or she will never get to participate in with their loved one now that he or she has AD/ADRD (i.e. travel, see their grandchildren grow up). Still, others indicated that unfinished business refers to unresolved hurts or closure and answers that one wasn’t able to get from the person they care for and now it is too late. Other responses ranged from unfinished business as a barrier standing in your way of being a good caregiver, practical tasks one is engaged in when not caring for their loved one (as minor as taking out the garbage to as major as preparing for a loved one’s death and getting affairs in order with the lawyer), and what the patient will never get to do now that he or she has AD/ADRD. One participant felt that stepping in and being able to care for his mom now that she is sick made his business feel finished rather than unfinished, as he felt caring for his mom is repaying her for all she did for him and filling a void in his life. Many referenced that they keep in mind that caregiving for someone with AD/ADRD is a temporary, albeit long, interruption in the business of life. Participants recommended that our team clarify what the study means by the term “unfinished business” and give examples.

**Table 2:** Demographics of *N* = 17 AD/ADRD caregivers engaged in usability and acceptability testing

Variable	M (SD) or *n* (%)
Age (years)	58.9 (9.53)
Gender	
Female	15 (88.2)
Male	2 (11.8)
Race	
White/Caucasian	10 (58.8)
Black/African American	4 (23.5)
Asian/Pacific Islander	3 (17.7)
Ethnicity	
Hispanic/Latino	3 (17.6)
Non-Hispanic/Non-Latino	14 (82.4)
Marital Status	
Married/Partnered	14 (23.5)
Single/Never Married	3 (17.6)
Relationship to patient	
Daughter of patient	11 (64.7)
Spouse of patient	4 (23.5)
Close family friend	1 (5.9)
Granddaughter of patient	1 (5.9)
Length of caregiving (years)	5–6
Time spent caregiving per day (hours)	8.5

**Table 3:** Ten discreet tasks (*N* = 16)

1. Navigate to “what is meaning centered psychotherapy?”
2. Click on “what will be discussed”
3. Go to “sessions”
4. Go to “session 4”
5. Watch the video for “session 4”
6. Go to “session 5”
7. Watch the video for “session 5”
8. Watch the video for “session 6”
9. Navigate back to “welcome”
10. Log out

**Table 4:** Results of the system usability scale (*N* = 16)

Item	Mean	Stand. deviation
1. I think that I would like to use this website frequently.	2.06	1.06
2. I found the website unnecessarily complex.	1.44	0.51
3. I thought the website was easy to use.	1.38	0.50
4. I think that I would need the support of a technical person to be able to use this website.	1.50	0.63
5. I found the various functions in this website were well-integrated.	1.63	0.62
6. I thought there was too much inconsistency in this website.	1.81	0.66
7. I would imagine that most people would learn to use this website very quickly.	1.81	0.83
8. I found the website very cumbersome to use.	1.63	0.81
9. I felt very confident using the website.	1.81	0.83
10. I needed to learn a lot of things before I could get going with this website.	1.81	0.98

Note: All 10 items were rated on a 5-point Likert scale ranging from 1 = strongly agree to 5 = strongly disagree. Negatively worded items 2, 4, 6, 8, and 10 were reverse coded and means were calculated across all participants for each of the 10 items, with lower means indicating more favorable usability of RELOAD-C.

**Table 5:** Comfort using Microsoft Teams (*N* = 9)

Item	*N* (%)
Did the participant join Teams successfully?	On their own, no help needed, 9 (100) With help only, 0 (0) Could not join Teams at all, 0 (0)
Instruct the participant to use the raise hand function. Were they able to complete this task?	On their own, no help needed, 6 (66.67) Able to complete but needed help, 3 (33.33) Wasn’t able to complete at all, even with help, 0 (0)
Instruct the participant to switch the microphone off and then back on. Were they able to complete this task?	On their own, no help needed, 8 (88.89) Could complete it but needed help, 1 (11.11) Could not complete this task, even with help, 0 (0)

**Table 6:** General feedback on video scripts (*N* = 15)

Theme	Examples
Increase reference to loneliness	Caregivers commented that while there was plentiful discussion on meaning and purpose in life, there wasn’t enough mention of loneliness. Caregivers maintained that their caregiving journey feels very much like their journey alone, and that they can be in a house filled with other people and yet still feel lonely. Caregivers indicated that they cannot get out of the house to socialize because they have nobody they can trust to stay with their loved one.
Include more content related to grief	Caregivers recommended that scripts be edited to include a discussion on the stages of grief, and how in the case of caring for a loved one with AD/ADRD, you lose the patient twice, as AD/ADRD is like the death of a family member but they haven’t physically died. References to “the long goodbye” and feeling like a “living widow” were made. Caregivers expressed that unlike with other illnesses, you never receive good news, as the disease gets progressively worse.
Changes to language	Many caregivers expressed that use of easy-to-understand language would be helpful, or in cases where the language couldn’t be changed, add definitions of key terms, such as “experiential.” Other participants requested we refrain from the use of the word “intervention” because it sounds like caregivers’ behavior requires correction.
Increase practical advice	Caregivers requested that we add practical advice (re-arranging traditions so it fits the patients’ needs, go out to eat at restaurants with family bathrooms, take patients food shopping and let them push the cart) as well as education on managing a loved one who is exhibiting behavioral and psychological symptoms of AD/ADRD.
Expand on role reversal with AD/ADRD	“Mom has now become the child”

**Table 7:** General feedback on social worker’s script (*N* = 15)

Theme	Examples
Terminology used to refer to the group meetings	Caregivers felt misled by our use of the term “support group” and encouraged us to refer to the virtual meetings as “therapy groups” given their structure and that they are facilitated by a trained mental health professional.
Outline group logistics during first meeting	Participants felt it was important for the social worker to outline the logistics of the group, particularly at the first meeting, such as re-playing that week’s video, reminding attendees that the group will meet for 1 hour, that meetings are confidential, participants should be in a private space with their microphones muted and cameras turned on, as they are comfortable.
Goals of the group meetings	Many caregivers agreed that interactive discussion is the goal for these virtual meetings.

**Table 8:** Summary of meaning-centered psychotherapy videos

Video number	Content
1	Introduction to the concept of “meaning” Story of Viktor Frankl Overview of four sources of meaning (historical, attitudinal, creative, experiential) Times when life has felt meaningful to you Identity change since becoming a caregiver
2	Exploration of historical sources of meaning Past, present and future legacy or story of life Note memories from your past that made a lasting impact on who you are today The legacy you live today, including being a caregiver, will impact the legacy you give in the future
3	Introduction to attitudinal sources of meaning How you choose to think about your role as a caregiver can help you experience growth and positive emotions You can draw on experiences in the past where you overcame challenges and use those lessons when thinking about how to respond to the challenges of caregiving
4	Introduction to creative sources of meaning It takes courage to create a life fully when you are facing illness and are so aware of the finiteness of life Being responsible and responding to the life you have been given Unfinished business Existential versus neurotic guilt
5	Introduction to experiential sources of meaning Feeling connected through love, beauty, humor Experience life through your five senses can free you from present challenges Transcendence
6	Conclusion and wrap-up Summary of four sources of meaning Reflection about use of the four sources of meaning Hopes for the future

## Data Availability

De-identified data from this study will be made available (as allowable according to institutional IRB standards) by emailing the corresponding author.

## Implications

- **Practice:** RELOAD-C, a web-based intervention to reduce loneliness by increasing meaning and purpose in life using concepts adapted from meaning-centered psychotherapy, is deemed usable and acceptable by dementia caregivers.
- **Policy:** Policymakers who want to address the epidemic of loneliness should explore routes for implementing evidence-based loneliness-reduction interventions in a scalable way.
- **Research:** The next step in this program of research is a large-scale randomized controlled trial to evaluate the efficacy of RELOAD-C in reducing loneliness in dementia caregivers.

## Analytic Code Availability

There is no analytic code associated with this study.
